# ERK/Nrf2 pathway activation by caffeic acid in HepG2 cells alleviates its hepatocellular damage caused by *t*-butylhydroperoxide-induced oxidative stress

**DOI:** 10.1186/s12906-019-2551-3

**Published:** 2019-06-20

**Authors:** Sung-Yong Yang, Min Cheol Pyo, Mi-Hyun Nam, Kwang-Won Lee

**Affiliations:** 1 212 CJ Food Safety Hall, Institute of Life Science and Natural Resources, Korea University, Anam-Dong, Sungbuk-Gu, Seoul, 02841 South Korea; 2 Department of Biotechnology, College of Life Sciences and Biotechnology, Korea University, Seoul, 02841 South Korea; 3 Department of Ophthalmology, University of Colorado School of Medicine, Aurora, CO 80045 USA; 4 Present Address: Samsung Bioepis, Analytic Development Team, Incheon, 21987 South Korea

**Keywords:** Caffeic acid, *tert*-butyl hydroperoxide, Glutamate-cysteine ligase, ERK/Nrf2 pathway, Antioxidant response element

## Abstract

**Background:**

Several studies have found that caffeic acid (CA), a well-known phytochemical, displays important antioxidant and anti-cancer activities. However, no evidence exists on the protective effect and its mechanisms that CA treatment alone has against oxidative stress induced by *tert*-butyl hydroperoxide (*t*-BHP) in HepG2 cells.

**Methods:**

Hepatoprotective activities such as cell viability, mRNA expression, and report gene assay were measured using HepG2 cell. Three types of genes and proteins related with detoxification in liver were used for measuring the hepatoprotective effects. Statistical analysis was performed using one-way ANOVA test and differences among groups were evaluated by Tukey’s studentized range tests.

**Results:**

The present study indicate that treatment with CA up-regulates heme oxygenase-1 (HO-1) and glutamate-cysteine ligase (GCL) mRNA and protein expressions in a CA-dose-dependent manner. In addition, translocation of nuclear factor-E2 p45-related factor (Nrf2) from the cytoplasm to the nucleus and phosphorylation of extracellular signal-regulated kinase, ERK and c-Jun N-terminal kinase, JNK which have been shown to be involved in mitogen-activated protein kinases, MAPKs are significantly enhanced by CA treatment. Furthermore, in cell nuclei, CA enhances the 5′-flanking regulatory region of human antioxidant response element (ARE) and activates the ARE binding site.

**Conclusion:**

Therefore, CA proved to be a stimulant of the expression of detoxification enzymes such as HO-1, GCLC, and GCLM through the ERK/Nrf2 pathway, and it may be an effective chemoprotective agent for protecting liver damage against oxidative damage.

**Graphical abstract:**

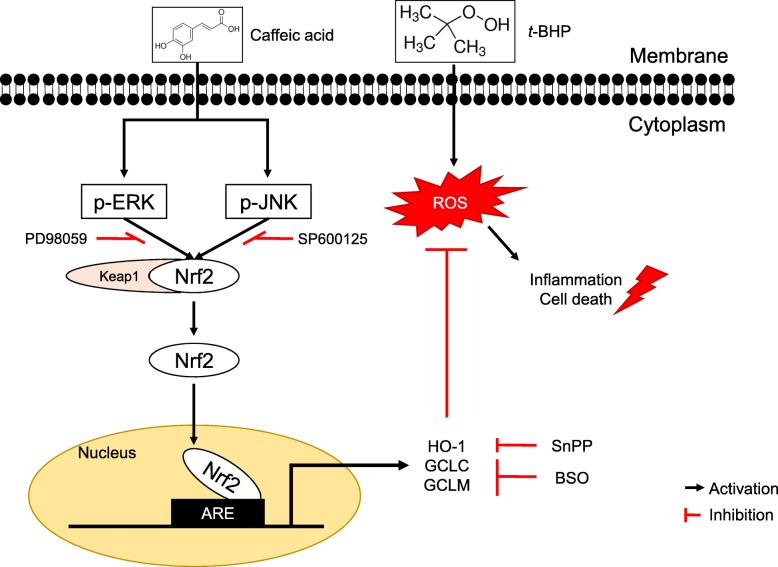

**Electronic supplementary material:**

The online version of this article (10.1186/s12906-019-2551-3) contains supplementary material, which is available to authorized users.

## Background

Results from many studies conducted worldwide demonstrate that numerous natural compounds could be useful as functional food ingredients, including those with antioxidant and/or anti-inflammatory activity [1, 2]. Caffeic acid (CA), the well-known phenolic compound widely present in plants, has shown a variety of pharmacological properties, such as anti-inflammatory, anti-cancer, and anti-viral activities [3]. Previously, we reported that CA present in perilla (*Perilla frutescens*) leaves plays an important role in increasing intracellular glutathione (GSH) content by stimulating glutamate-cysteine ligase (GCL) via c-Jun N-terminal kinase (JNK)/activator protein-1 (AP-1) pathway [4]. However, the role of CA in activating nuclear factor-E2 p45-related factor (Nrf2) involved in the antioxidant redox cycle associated with cell survival is not well studied.

*tert*-Butyl hydroperoxide (*t*-BHP) is used to induce oxidative stress in in vitro models and generates reactive oxygen species (ROS) that proceed to effect lipid peroxidation [5]. In addition, *t*-BHP can trigger apoptosis through the release of cytochrome c from mitochondria [6]. Recently, *t*-BHP was reported to down-regulate the expression of superoxide dismutase (SOD) in rats [7]. Therefore, it seems important to investigate the mechanism by which *t*-BHP-induced oxidative stress causes cytotoxicity.

Nuclear factor-E2 p45-related factor (Nrf2), which belongs to the Cap’n’Collar family of basic leucine zipper transcription factors, has been identified as a key species involved in antioxidant responsive element (ARE)-mediated gene expression [8]. Studies conducted in the past showed that the deletion of the Nrf2 gene in mice results in a decrease in the expression of the phase II detoxification genes, including those encoding heme oxygenase-1 (HO-1) and GCL [9, 10]. Mitogen-activated protein kinases (MAPKs) can be activated by a wide variety of different stimuli, in particular, extracellular signal-regulated kinase (ERK) is generally activated by growth factors. Recently, results from several studies indicated the presence of a relationship between phosphorylation of ERK and Nrf2 activation [11, 12].

Therefore, we aimed to investigate the protective effect of CA with respect to hepatic damage caused by *t*-BHP-induced oxidative stress, and we attempted to assess how CA affects Nrf2 translocation, and whether it is accompanied by MAPK activation under oxidative stress conditions.

## Methods

### Reagents and materials

Dulbecco modified eagle medium (DMEM), penicillin-streptomycin, trypsin-EDTA, Trizol®, lipofectamine 2000 transfection reagent, and bovine serum albumin were purchased from Life Technologies (Grand Island, NY, USA), whereas caffeic acid, 3-(4,5-dimethylthiazol-2yl)-2,5-diphenyl tetrazolium bromide (MTT), DMSO, *t*-BHP, and 2,4-dinitrofluorobenzene (FDNB) were purchased from Sigma-Aldrich (St. Louis, MO, USA). Antibodies against ERK1/2, JNK, phospho-ERK, and GCL were purchased from Santa Cruz Biotechnology (Dallas, TX, USA), and those against p38, phosphorylated-JNK, and phosphorylated-p38 were purchased from Cell Signaling Technology (Danvers, MA, USA). pGL4.37 (Luc2P/ARE/Hygro) and the dual-luciferase reporter assay kit were purchased from Promega (Madison, WI, USA). A BCA protein assay kit was purchased from Thermo Scientific (Waltham, MA, USA).

### Cell culture and viability assay

Human hepatoma HepG2 cells were obtained from the ATCC (Manassas, VA, USA) and were cultured in DMEM containing 1 g/L glucose, 10% (v/v) FBS, 3.7 g/L sodium bicarbonate, 100 U/mL penicillin and streptomycin. Cells were maintained at 37 °C in a humidified atmosphere containing 5% CO_2_. HepG2 cells were seeded at a density of 5 × 10^5^ cells/well in 6-well plates to the amount of selected mRNAs, and the expression level of target protein expression. Cells were seeded at a density of 5 × 10^4^ cells/well in a 24-well plate to conduct the cytotoxicity and reporter gene assays. In addition, to conduct the ROS assay. Cells were grown in DMEM containing 10% FBS but were transferred to serum-free medium 20 h before the assays were conducted. After cell attachment, the cells were washed two times with phosphate-buffered saline (PBS) solution, incubated with CA (at a concentration of 20, 10, or 5 μM) for 24 h, and subsequently, were washed two times with PBS solution and then 0.3 mM *t*-BHP for 2 h. Cells were then transferred to DMEM containing 5 mg/mL of MTT, where they were incubated for 4 h at 37 °C. The medium was then removed and 200 μL of DMSO was added to each well to dissolve the formazan crystals. Finally, the optical density was measured at a wavelength of 540 nm.

### Isolation and culture of primary rat hepatocytes

Hepatocytes were isolated from 7 week old male Wistar rats weighing 200–250 g. Rat hepatocytes were prepared by the collagenase perfusion method, and several modifications were made to the previously described methods [13, 14]. This experiment was carried out according to the guidelines of the Committee for Ethical Usage of Experimental Animals of Korea University (KUIACUC-20100319-2). The medium was replaced 4 h after plating and then cultured in a humidified incubator in airy atmosphere at 37 °C.

### Analysis of intracellular ROS

For determining intracellular ROS, HepG2 cells were exposed to 100 μM dichlorofluorescein diacetate (DCFH-DA) for 30 min at 37 °C. After being washed two times with PBS solution, these cells were exposed to various concentrations of CA dissolved in phenol-red-free DMEM medium for 24 h, washed two times with PBS solution, and then treated with *t*-BHP for 2 h. The fluorescence of 2′7′-dichlorofluorescein was detected at an excitation wavelength of 485 nm and an emission wavelength of 535 nm using a multi-plate reader (Sense; HIDEX, Turku, Finland).

### Quantitative PCR (qRT-PCR) and RT-PCR

Total RNA (0.5 μg) collected using the Trizol reagent was reverse-transcribed into cDNA using a cDNA synthesis kit (Legene Biosciences, San Diego, CA, USA). The following primers for RT-PCR and qRT-PCR were designed based on the published cDNA sequences (Additional file 1: Table S1). RT-PCR was conducted in a 20 μL solution according to the manufacturer’s protocol (DreamTaq DNA polymerase, Thermo Scientific, Pittsburgh, PA, USA). qRT-PCR was performed using the real-time SYBR Green method on a BioRad iQ-5 thermal cycler, and PCR was conducted in a 20 μL of solution according to manufacturer’s instructions (iQ SYBR Green Supermix, Bio-rad, Hercules, CA, USA).

### Luciferase reporter assay

HepG2 cells were seeded onto 24-well plates 24 h before transfection at a density of 1 × 10^5^ cells/well. These cells were then transfected with a pGL4.37 luciferase plasmid (luc2p/ARE/Hygro) using Lipofectamine 2000 (Life Technologies) according to manufacturer’s instructions. After 4 h incubation at 37 °C, the transfection medium was replaced with complete medium, and cells were incubated for an additional 24 h. After that, the cells were treated with CA for 24 h and then treated with *t*-BHP for 2 h. Next, the luciferase assay was performed using a dual-luciferase assay kit (Promega). Luciferase activity values were then quantified with a luminometer, and the values of firefly- and renilla-luciferase expression were normalized to the luciferase activities of untreated cells.

### Preparation of cytosolic and nuclear proteins

HepG2 cells were incubated in the presence of CA in growth medium for 24 h and then treated with *t*-BHP for 2 h. Cells were washed with ice-cold PBS solution, harvested by scrapping, spun down at 12,000 rpm for 5 min at 4 °C, and re-suspended in cytosolic extract buffer (10 mM Hepes, 10 mM KCl, 0.1 mM EDTA, 1 mM DTT, 1 mM PMSF, 0.8% NP40, 5 μg/mL of leupeptin, and aprotinin, pH 7.8). After mixing, cells were centrifuged at 12,000 rpm for 2 min at 4 °C, and the supernatant (cytosolic extract) was collected. The cell pellets were then re-suspended in nucleic extract buffer (50 mM Hepes, 50 mM KCl, 300 mM NaCl, 0.1 mM EDTA, 1 mM DTT, 1 mM PMSF, 20% glycerol, 5 μg/mL of leupeptin, and aprotinin, pH 7.8). After the cell suspension was vigorously mixed for 10 min at 4 °C, it was centrifuged at 12,000 rpm for 2 min at 4 °C, and the supernatant (nuclear extract) was collected. Protein concentration was determined by BCA protein assay reagent.

### Western analysis

HepG2 cells were grown in 6-well plates and treated with CA. Protein samples were separated on 10% SDS-polyacrylamide gels and electrotransferred to Immobilon-P transfer membranes (Millipore, Billerica, MA, USA). Immunoblotting was performed using antibodies against GCL, MAPKs, and phosphor-MAPKs. The protein bands were detected using an Enhanced Chemiluminescence Detection kit (Abclon, Seoul, Korea). Data are expressed as fold-induction of treated samples with respect to the vehicle control.

### Preparation of nuclear extract and electrophoretic mobility shift assay (EMSA)

HepG2 cells were incubated with CA in growth medium for 24 h. Nuclear proteins were isolated using the NE-PER™ nuclear and cytoplasmic extraction kit (Pierce, Rockford, IL, USA), and EMSA was conducted using the Lightshift® Chemiluminescent kit (Pierce, Rockford, IL, USA) according to manufacturer’s instructions. The Nrf2 probe sequence 5′-TCA GCG ACT GGG ACT TTT CT-3′ was obtained from a commercial source (Cosmo Genetech, Seoul, Korea), and it contained the Nrf2 binding site with a 5′ biotin label. The binding reactions were carried out for 30 min, and the relevant reaction mixture contained 5 μg of nuclear proteins. To determine the binding specificity to the oligonucleotide, a 200-fold excess of unlabeled Nrf2 was added to the extract from CA-treated cells. DNA–protein complexes were separated under non-denaturing conditions on a 6% polyacrylamide gel using 0.5X TBE (45 mM Tris, pH 7.5, 45 mM boric acid, 2 mM EDTA) as a running buffer. The results were recorded using a Chemiluminescent Nucleic Acid Detection Module kit (Pierce, Rockford, IL, USA).

### Statistical analysis

Statistical analysis was performed using SAS ver. 9.3 (SAS Institute, Cary, NC, USA). Parameter values were expressed as mean ± standard deviation. Differences among groups were evaluated by one-way analysis of variance and Tukey’s studentized range tests. Differences characterized by a *p*-value under 0.05 were considered significant.

## Results

### Protective effect of CA on *t*-BHP-induced oxidative stress

**Fig. 1:**
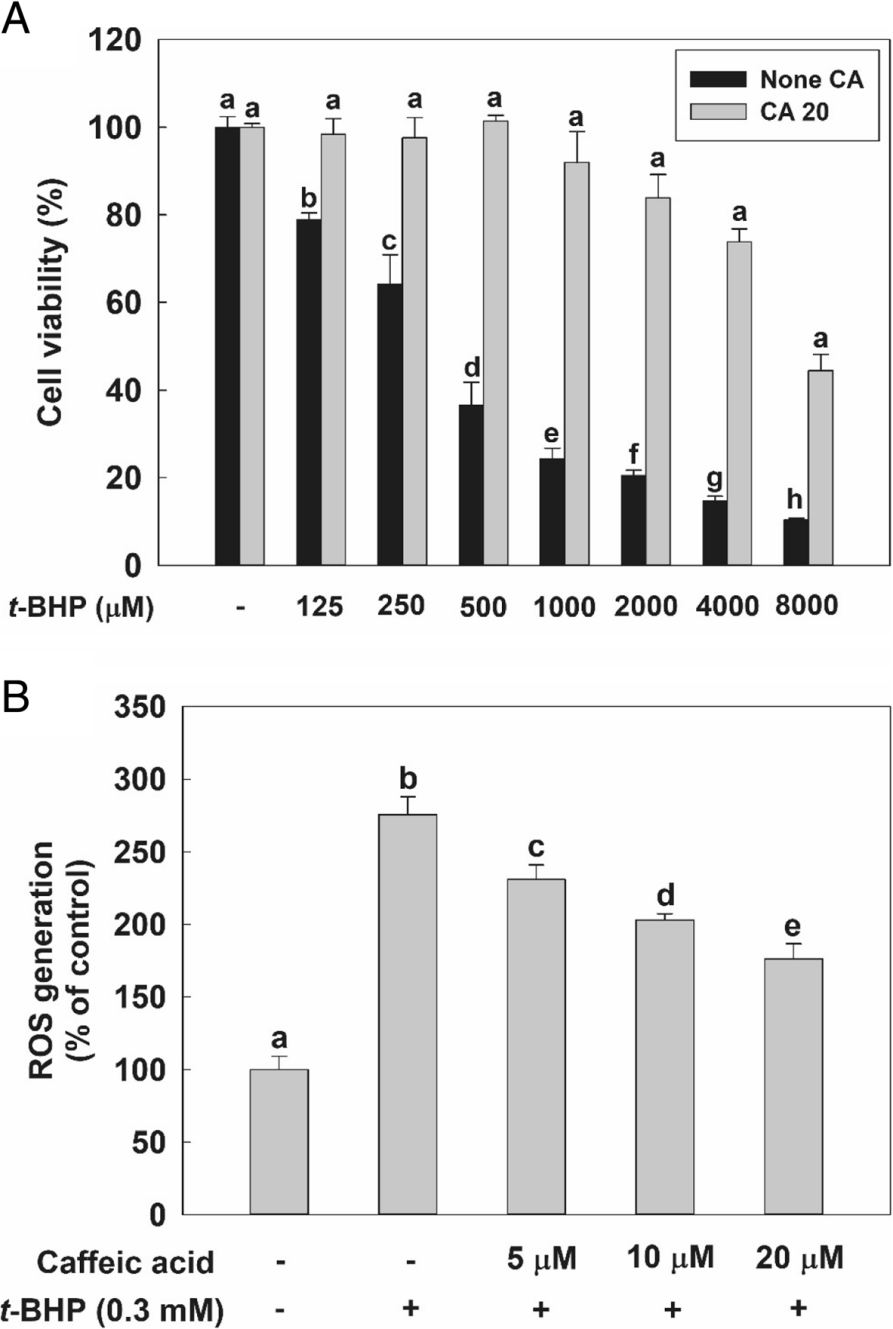
Effect of Caffeic acid (CA) treatment on HepG2 cells experiencing *tert*-butyl hydroperoxide (*t*-BHP)-induced oxidative cytotoxicity. **a** Cells were seeded at a density of 5 × 10^4^ cells/well in a 24-well plate. Viability of cells treated with 20 μM of CA for 24 h or not treated with this compound, before having been treated with various concentrations of *t*-BHP for 2 h. **b** Reactive oxygen species (Hsieh, #32) generation by cell cultures as a percentage of the generation of the control mixture. HepG2 cells were seeded at a density of 1 × 10^4^ cells/well in a 96-well plate. After seeding, cell were preincubated with 100 μM of dichlorofluorescin diacetate (DCFH-DA) for 30 min at 37 °C. They were then exposed to either 0 or 0.3 mM concentrations of *t*-BHP and to various concentration of CA (0–20 μM). ROS generation values are expressed as mean ± standard deviation (*n* = 3). Different letters indicate signification differences at *p* < 0.05 by Tukey’s studentized range tests

As can be seen from the first set of bars of the diagram in Fig. 1a, the value for the viability of HepG2 cells treated with 20 μM CA was more than 95%, indicating that CA is non-cytotoxic to these cells in this concentration. Oxidative stress induced by various concentrations (0 to 8 mM) of *t*-BHP decreased cell viability in a dose-dependent manner after a 2 h incubation. Under the same conditions of oxidative stress, the pretreatment with 20 μM CA significantly enhanced cell viability with respect to the case of cells incubated with the same concentration of *t*-BHP, but without having undergone the pretreatment with CA. Furthermore, HepG2 cells exposed to 250 and 500 μM concentrations of *t*-BHP (in the absence of CA pre-treatment) showed about 60 and 40% of cell viability with respect to the no-*t*-BHP case.

As can be seen in Fig. 1b, exposing HepG2 cells to a 0.3 mM concentration of *t*-BHP caused the amount of intracellular ROS to increase to about 270% of the level of the control. However, the pre-treatment with CA significantly decreased the amount of intracellular ROS generated in a dose-dependent manner. Therefore, we used a 0.3 mM concentration of *t*-BHP to induce oxidative stress in HepG2 cells and a maximum 20 μM concentration of CA for further in vitro studies, such as those aimed at probing enzyme activity and signaling pathways.

### Effect of CA on gene expression in HepG2 cells and rat primary hepatocytes

**Fig. 2:**
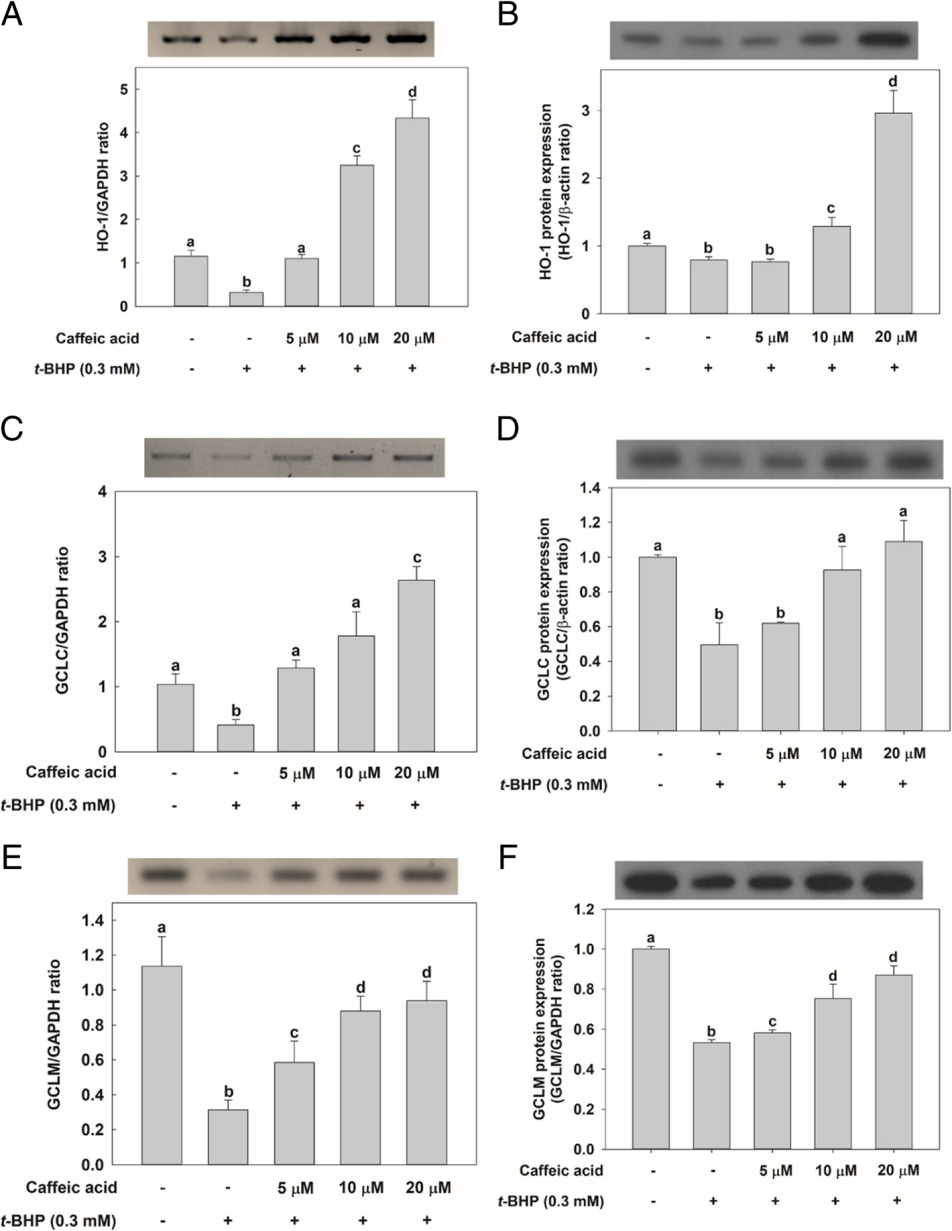
Effect of CA treatment on the mRNA levels of specific genes or on the expression of the protein they encode in HepG2 cells. Except for the control, in which cells were not exposed to any chemical, cells were incubated with CA at different concentrations (5, 10, and 20 μM) — or not at all — for 24 h before being incubated with 0.3 mM *t*-BHP for 2 h. Total RNA was extracted using the Trizol reagent and an equivalent amount of RNA was converted into cDNA using the reverse transcriptase kit implementing manufacturer’s instructions. RT-PCR and qRT-PCR experiments were ultimately performed to analyze the amount of **a** HO-1, **c** glutamate-cysteine ligase (GCL) catalytic unit (GCLC), and **e** GCL modifier subunit (GCLM) mRNA in HepG2 cells. Proteins (10 μg) were separated by 10% SDS-PAGE and electro-transferred to a polyvinylidene difluoride (PVDF) membrane. Immunoblotting was performed using monoclonal or polyclonal antibodies against **b** heme oxygenase-1 (HO-1), **d** GCLC, and **f** GCLM. Values are expressed as mean ± standard deviation (*n* = 3). Different letters indicate signification differences at *p* < 0.05 by Tukey’s studentized range tests

**Fig. 3:**
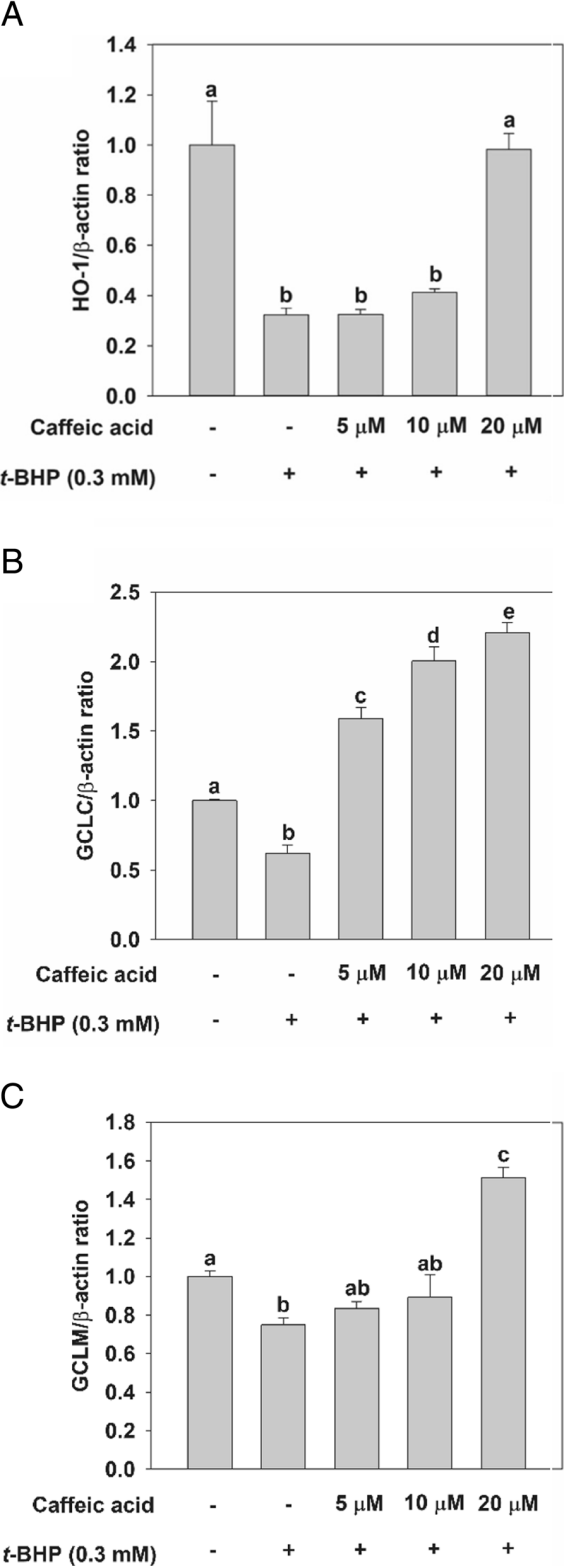
Effect of CA treatment on the mRNA levels of specific genes they encode in rat primary hepatocytes. Except for the control, in which cells were not exposed to any chemical, cells were incubated with CA at different concentrations (5, 10, and 20 μM) — or not at all — for 24 h before being incubated with 0.3 mM *t*-BHP for 2 h. Total RNA was extracted using the Trizol reagent and an equivalent amount of RNA was converted into cDNA using the reverse transcriptase kit implementing manufacturer’s instructions. qRT-PCR experiments were ultimately performed to analyze the amount of **a** HO-1, **b** GCLC, and **c** GCLM mRNA in rat primary hepatocytes. Values are expressed as mean ± standard deviation (*n* = 3). Different letters indicate signification differences at *p* < 0.05 by Tukey’s studentized range tests

Then, we investigated the effect of CA treatment on the expression of genes associated with antioxidant activities, such as HO-1 and GCL. First, RT-PCR was used to analyze mRNA levels in HepG2 cells treated with CA. Although a down-regulation of GCLC, GCL modifier subunit (GCLM), and HO-1 mRNA levels were observed as a consequence of treatment with *t*-BHP, cells in the CA-treatment groups displayed an increase in HO-1 and GCLC mRNA levels in spite of *t*-BHP treatment compared with cells in the control group without CA-treatment (Fig. 2a, and c). Especially, GCLM mRNA expression also recovered as a consequence of CA pretreatment in a dose-dependent manner although it did not show as much as GCLC and HO-1 gene expressions (Fig. 2e). Similar to the results of hepG2 cells, the mRNA expression of HO-1, GCLC, and GCLM in the CA-treated groups increased despite the *t*-BHP treatment in rat primary hepatocytes (Fig. 3a-c). In all cases, gene expressions increased in cells in the CA-treatment groups compared with cells in the group treated only with *t*-BHP. Furthermore, all increases in gene expressions observed as a consequence of CA treatment were dose-dependent. In addition, the protein expressions of the HO-1 and GCL families in cells in the CA treatment groups were all increased compared with those in cells in the group treated only with *t*-BHP (Fig. 2b, d, and f).

### Effect of CA on nuclear translocation of Nrf2

**Fig. 4:**
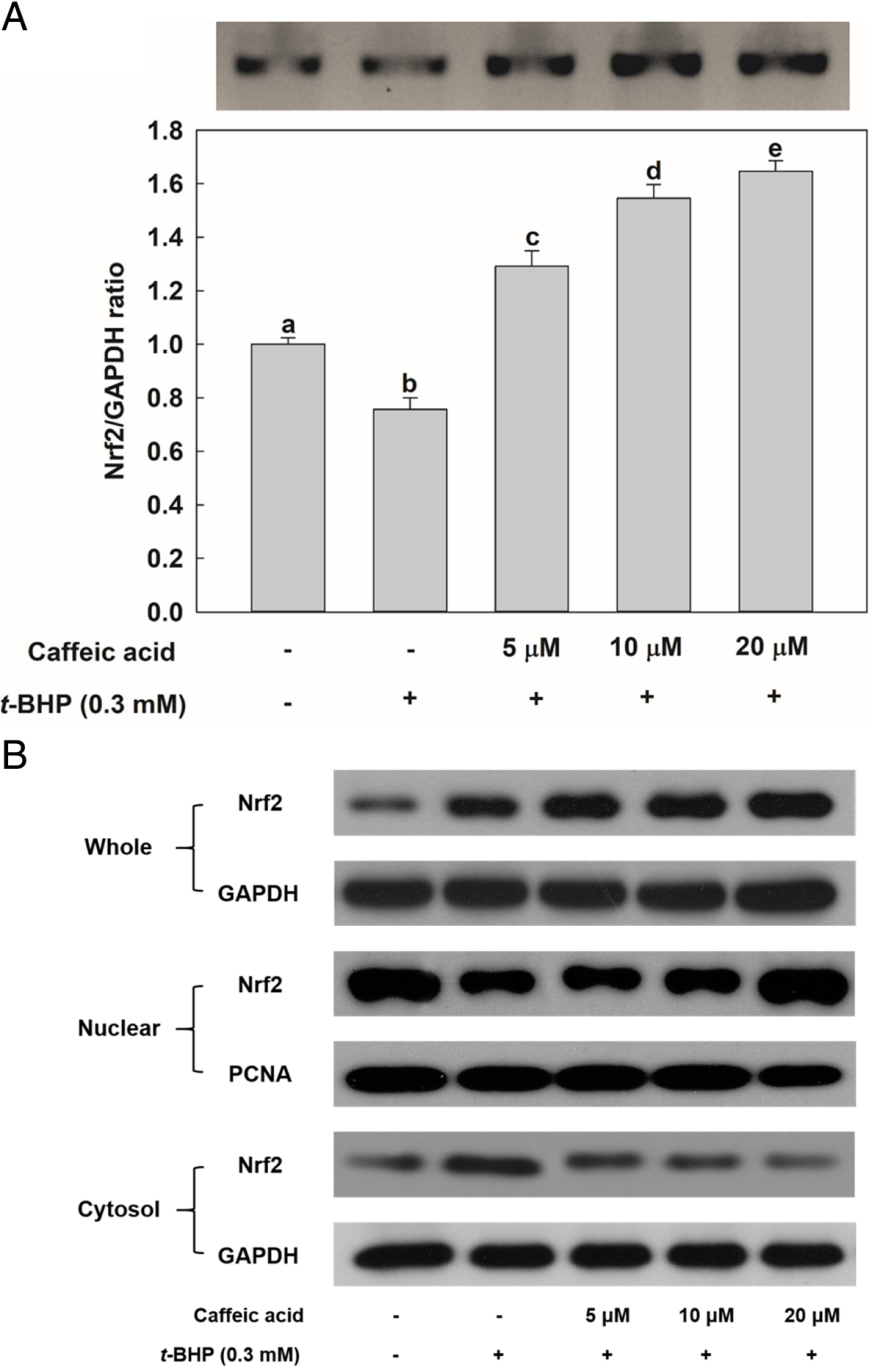
Effect of CA treatment on nuclear factor-E2 p45-related factor (Nrf2) gene expression and on protein nuclear translocation in HepG2 cells. Except for the control, in which cells were not exposed to any chemical, cells were incubated with CA (5, 10, and 20 μM) — or not at all — for 24 h and before being incubated with 0.3 mM *t*-BHP for 2 h. **a** Total RNA was extracted using the Trizol reagent and equivalent amount of RNA was converted into cDNA with the reverse transcriptase kit according to manufacturer’s instructions. RT-PCR and qRT-PCR experiments were performed to determine the amount of Nrf2 mRNA in HepG2 cells. **b** Proteins (10 μg) were separated by 10% SDS-PAGE and electro-transferred to PVDF membrane. Values are expressed as mean ± standard deviation (*n* = 3). Different letters indicate signification differences at *p* < 0.05 by Tukey’s studentized range tests

Nrf2 has been identified as a key transcription factor involved in ARE-mediated gene expression [8]. The results demonstrated that Nrf2 gene expression increased as a result of CA treatment (Fig. 4a). Nrf2 mRNA level decreased in HepG2 cells as a result of *t*-BHP treatment. However, the cells pre-treated with CA dose-dependently increased the level of Nrf2 mRNA. As can be seen in Fig. 4b, nuclear translocation of Nrf2 was also observed by Western analysis. Treatment with *t*-BHP reduced the amount of Nrf2 protein observed in HepG2 cell nuclei; conversely, a larger amount of Nrf2 was observed in the cytosolic fraction of *t*-BHP-treated cells than in the cytosolic fraction of control cells. In spite of *t*-BHP treatment, the cells subjected to pretreatment with CA experienced an up-regulation of whole Nrf2 protein synthesis and of the nuclear translocation of this protein, compared to cells subjected only to the effect of *t*-BHP-induced oxidative stress. HepG2 cell treatment with 20 μM CA resulted in the translocation of the Nrf2 protein to the nucleus. Therefore, our results indicate that the increase in the level of HO-1 and GCL subunit mRNAs observed in CA-treated cells was associated with the translocation of Nrf2 to cell nuclei.

### Effect of CA on MAPK phosphorylation

**Fig. 5:**
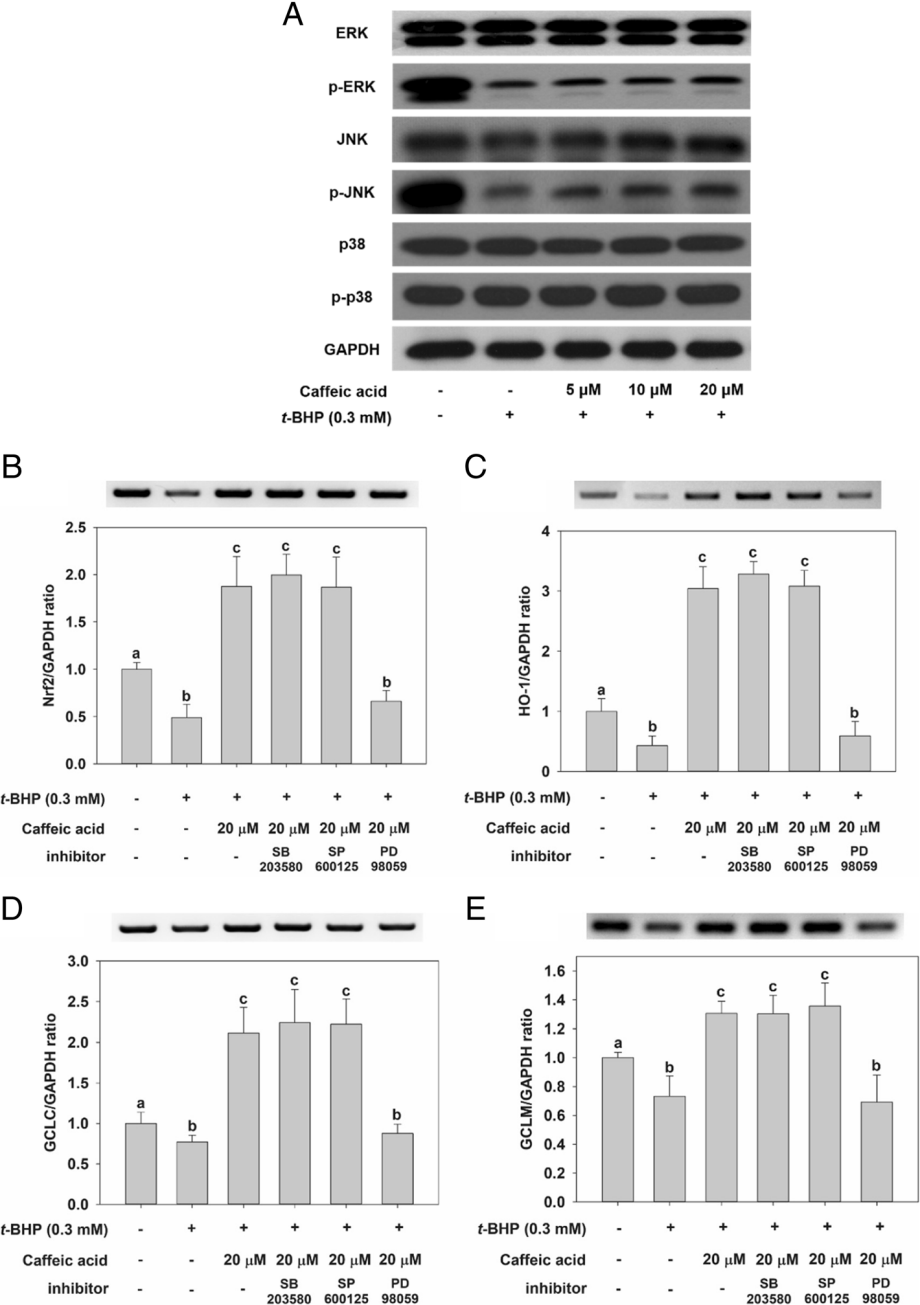
Effect of CA treatment on total expression and phosphorylation levels of ERK, JNK, and p38 in HepG2 cells. **a** Except for the control, in which cells were not exposed to any chemical, cells were incubated with CA (5, 10 and, and 20 μM) for 24 h, before being incubated with 0.3 mM *t*-BHP for 2 h. Cells were pretreated with a 10 μM concentration of MAPK-specific inhibitors for 1 h and then treated with 20 μM CA for 24 h. Cells were washed, and treated with 0.3 mM *t*-BHP for 2 h. RT-PCR and qRT-PCR experiments were performed to determine the amount of **b** Nrf2, **C** HO-1, **d** GCLC, and **e** GCLM mRNA found in HepG2 cells. Values are expressed as mean ± standard deviation (n = 3). Different letters indicate signification differences at *p* < 0.05 by Tukey’s studentized range tests

The effect of CA treatment on MAPK phosphorylation in HepG2 cells was examined by incubating the cells with CA for 24 h (Fig. 5a). No significant difference in the phosphorylation of p38 was observed with incubation with *t*-BHP. However, treatment with *t*-BHP significantly decreased phosphorylation of the ERK and JNK proteins. The pretreatment of cells with CA increased phosphorylation of ERK and JNK. These results indicated that *t*-BHP-induced oxidative stress down-regulates JNK and ERK phosphorylation, leading to affect cell viability. However, the treatment with CA could be protective against *t*-BHP-induced liver damage via up-regulation of JNK and ERK phosphorylation. To clarify how the expression of genes associated with antioxidant activity is affected by the phosphorylation pathway of JNK and ERK, HepG2 cells were pretreated with the specific inhibitors for ERK1/2 (PD98059) and JNK (SP600125) as well as p38 (SB203580). As can be seen from the data in Fig. 5b–e, *t*-BHP-induced oxidative stress down-regulates the expression of genes associated with antioxidant activity, such as HO-1, GCLC, GCLM, and Nrf2, as measured by conducting RT-PCR and qRT-PCR experiments. However, the pretreatment with CA of cells exposed to *t*-BHP led to an almost complete recovery of mRNA expression, with respect to the control. Notably, the pretreatment with PD98059 was associated with a reduction in HO-1, GCLC, GCLM, and Nrf2 mRNA levels. By contrast, pretreatment with SB203580 and SP600125 was not associated with significant differences with respect to the no-inhibition case (Fig. 5b–e).

### Effect of CA on the viability of cells pretreated with specific inhibitors

**Fig. 6:**
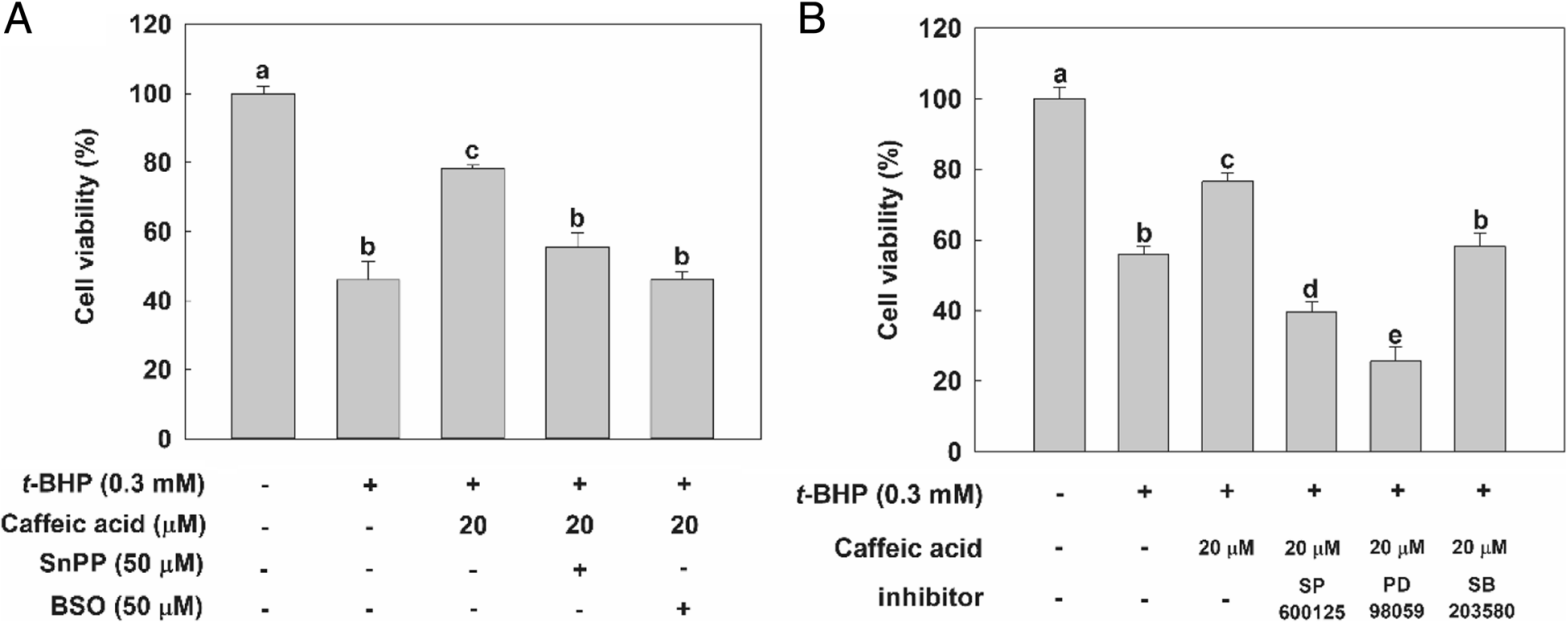
Cytoprotective effect of CA treatment on HepG2 cells pretreated with (**a**) HO-1- and GCL-specific inhibitors; and (**b**) JNK-, ERK, and p38-specific inhibitors. Except for the control, in which cells were not exposed to any chemical or inhibitor, cells were either pretreated with 50 μM of HO-1- or GCL-, or 10 μM of JNK-, ERK or p38-specific inhibitors for 1 h — or not exposed to an inhibitor at all— then treated with 20 μM CA for 24 h —or not exposed to CA at all. Cells were washed twice with PBS solution and treated with 0.3 mM *t*-BHP for 2 h. Cell viability was measured implementing the MTT assay. Values are expressed as mean ± standard deviation (n = 3). Different letters indicate signification differences at *p* < 0.05 by Tukey’s studentized range tests

To confirm the cytoprotective effect of CA through the possible mechanisms that affect accumulation of HO-1 and GCL enzymes, HepG2 cells were pre-incubated with specific inhibitors, such as SnPP (for HO-1), BSO (for GCL), SP600125 (for JNK), PD98059 (for ERK), and SB203580 (for p38). As can be evidenced from the data in Fig. 6a, when HO-1 and GCL were blocked by their specific inhibitors, treatment with CA did not increase the viability of cells exposed to *t*-BHP. Interestingly, although exposure to SP600125 and PD98059 reduced the viability of cells treated with CA and *t*-BHP, inhibition of p38 had no effect on cell viability (Fig. 6b). Hence, these results indicate that CA protects HepG2 cells against *t*-BHP-induced oxidative stress by up-regulating HO-1, GCLC and GCLM via ERK signaling pathway-mediated Nrf2 gene expression, and JNK pathway as well.

### Measuring the effect of CA on Nrf2 activation through the reporter gene assay

**Fig. 7:**
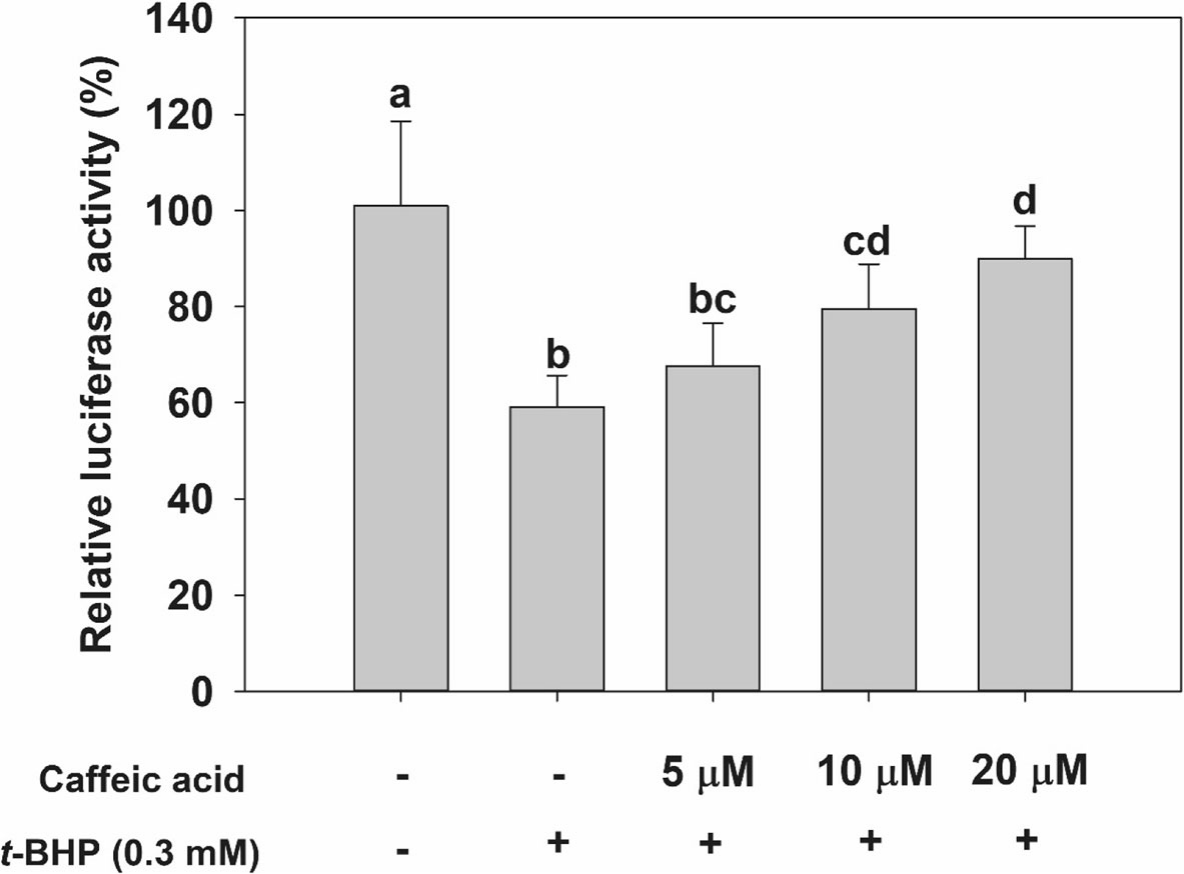
Effect of CA treatment on 5′-flanking regulatory region of antioxidant response element (ARE) in HepG2 cells. HepG2 cells were transfected with the pGL4 luciferase plasmid (luc2p/ARE/Hygro). They were then treated with various concentrations of CA—or not at all—and 0.3 mM of *t*-BHP. Luciferase activity was then measured in HepG2 cell lysates. Values are expressed as mean ± standard deviation (n = 3). Different letters indicate signification differences at *p* < 0.05 by Tukey’s studentized range tests

To investigate the mechanism by which CA effects hepatoprotection against *t*-BHP-induced oxidative stress, we measured the luciferase activity about 5′-flanking regulatory region of human ARE. Transient transfection analysis was performed using the pGL4.37 vector containing four copies of the ARE gene that encodes a protein driving transcription of the luciferase reporter gene *luc2P*. Treatment with *t*-BHP decreased luciferase activity by about 60% compared with the untreated cells in the control; however, pretreating cells with 20 μM of CA led to a significant recovery of the activity to about 89% of the no-treatment control (Fig. 7). These results show that CA treatment can affect Nrf2 binding to the ARE, ARE-mediated gene expression, and induction.

### Effect of CA on ARE binding site activation

**Fig. 8:**
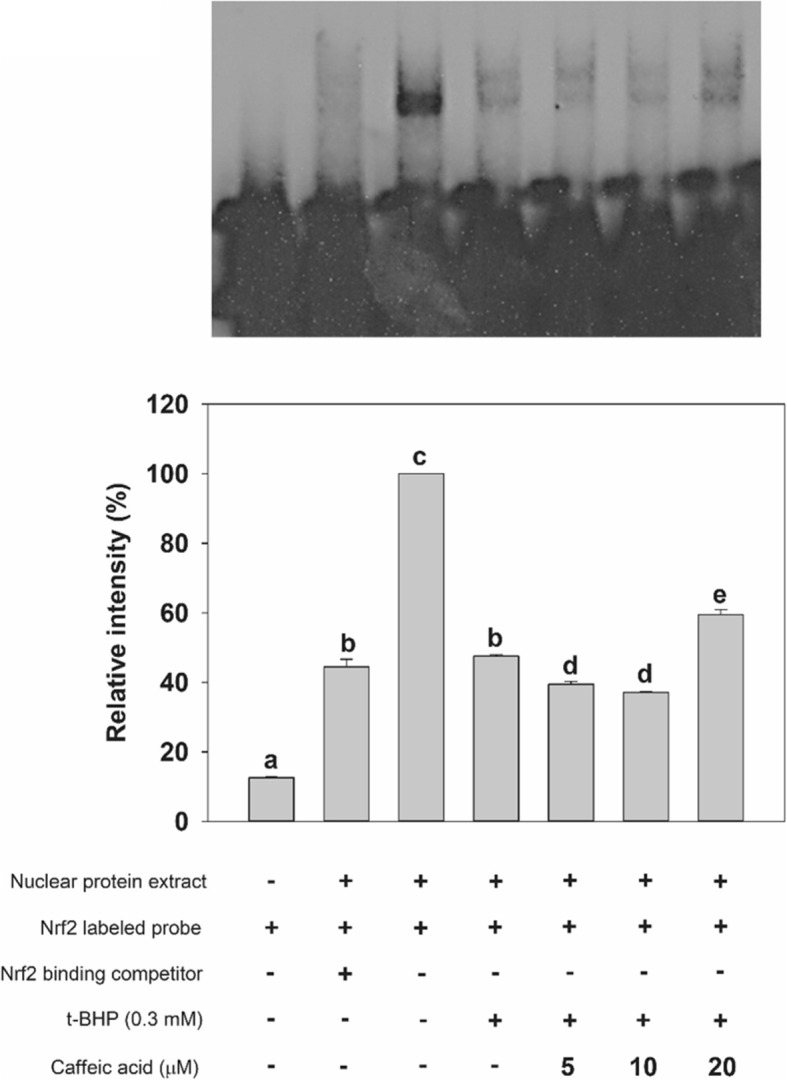
Effect of CA treatment on DNA-binding activity of ARE in HepG2 cells. Cells were treated with 0.1% DMSO (lanes 2 and 4) and with the indicated concentration of CA (lanes 5–7) for 24 h. After treatment with CA, HepG2 cells were incubated with a 0.3 mM concentration of *t*-BHP for 2 h. Nuclear proteins (8 μg) were isolated, incubated with a labeled ARE sequence and subjected to electrophoretic mobility shift assay. A competition experiment (200-fold excess of unlabeled ARE) was performed with nuclear protein extracted from the non-treated control (lane 2)

EMSA was performed on nuclear extracts of non-treated HepG2 cell controls as well on cells treated with *t*-BHP and/or CA. Cells were treated with the 5, 10, and 20 μM of CA for 24 h and their nuclear extracts were subsequently assayed. The nuclear extracts of *t*-BHP-treated cells were used as an oxidative stress control. Treatment with 0.3 mM *t*-BHP was associated with a down-regulation in ARE DNA-binding ability (Fig. 8, lane 4) compared with the non-treated control (Fig. 8, lane 3). The pretreatment with CA at 20 μM concentration caused an up-regulation in DNA-binding complex of Nrf2 with respect to the case of cells in the oxidative stress control (Fig. 8, lane 7). The specificity of the band was confirmed by performing a competition assay using a sample containing a 200-fold excess of the unlabeled ARE probe (Fig. 8, lane 2). Treatment with *t*-BHP affected the ARE binding site activation, and exposing to CA, *t*-BHP-treated cells led to a recovery of the ARE activation.

## Discussion

Caffeic acid (CA), a natural phenolic compound widely present in plants, has been investigated in the past for its protective effect against chemically induced intracellular oxidative damage [15–17]. Our group has studied this compound for its antioxidant and hepatoprotective activities [18, 19]. Although we did not similarly structured phenolic acids in the present study, we previously reported that CA in the presence of rosmarinic acid, is responsible for the protective potency of perilla leaf extract on *t*-BHP induced oxidative stress [18]. Our group showed that aqueous extracts of *Perilla frutescens* leaves protected hepatocytes against *t*-BHP-induced toxicity [20]. Also, perilla leaf extract and CA from the extract up-regulated GSH synthesis in HepG2 cells and rat liver tissue, via activation of the transcription factor AP-1 [4, 21]. In addition, perilla leaf extract activated nuclear translocation of cytosolic Nrf2 and increased HO-1 gene expression [22]. Although results from several studies have indicated that CA protects from chemically induced cellular damage in vitro [23, 24], to the best of our knowledge, the hepatoprotective effect of CA against *t*-BHP-induced oxidative stress via MAPKs and Nrf2 activation had not been previously investigated. Thus, the present study was investigated to provide possible mechanisms that CA treatment has against *t*-BHP-induced oxidative stress in liver cells. In addition, it is worth mentioning that *t*-BHP was used as an oxidative agent in this study. Because *t*-BHP is not relevant to human exposure, it may be appropriate to test other oxidative stress agents to human that may be exposed to humans for future experiments.

To survive under a variety of environmental stresses, hepatocytes retain a cellular defense systems that protects them against oxidative challenges [25, 26]. One of these system requires phase II drug-metabolizing enzymes, such as glutathione-S-transferase and UDP-glucuronosyltransferase [27], and antioxidant enzymes, such as HO-1, NADP(H):quinone oxidoreductase-1 (NQO-1), and GCL [28, 29]. Our previous study reported that CA treatment only increased only GCL catalytic subunit, GCLC mRNA level in normal phase cell [4]. However, as can be evinced from the data in the present study, cell treatment with CA led to a dose-dependent significant increase in the expression of not only GCLC but also GCLM, compared with cells treated only with *t*-BHP. These discrepancies may be due to the concentration of CA treated in the cells, and/or the incubation time treated in the CA in the presence or absence of *t*-BHP. In the previous experiment [4], HepG2 cells were treated with a concentration of CA from 62 μM up to 250 μM for 8 h without *t*-BHP treatment, whereas the maximum concentration of CA used in this experiment was 20 μM for 24 h followed by *t*-BHP treatment for 2 h. On the other hand, the L-02 liver cells which were incubated with CA (10 and 50 μM) for 15 min, and then incubated with 7.5 mM acetaminophen for 48 h had no effect on GCLC and GCLM mRNA/protein [30]. Huang et al. reported that up-regulated the mRNA/protein expression of GCLC and GCLM was observed in rat primary hepatocytes treated with flavones including 25 μM chrysin and apigenin for 24 h [31]. Treatment of RAW264.7 cells with *t*-BHP significantly reduced GCLC and GCLM mRNA levels, and treatment of these cells with 25 μM licochalcone A, a natural phenol for 18 h, led to the recovery of both GCLC and GCLM gene expression levels [32].

Our results demonstrated that cytotoxicity caused by *t*-BHP-induced oxidative stress was recovered by CA treatment by way of the up-regulation of the expression of detoxifying enzymes like HO-1, GCLC, and GCLM. These enzyme-encoding genes, whose expression is associated with detoxification activity, were regulated by a consensus *cis*-element located at the 5′-flanking promoter region, such as the antioxidant response element (ARE) [33]. The transcription factor Nrf2 plays a key role in the antioxidant redox cycle associated with cell survival, because it is an essential component of the ARE-binding transcription factor [8]. Investigating Nrf2 translocation, we observed that cells treated with CA experienced a significant and dose-dependent nuclear accumulation of Nrf2. On the other hand, in cells treated with CA was observed a reduction in the amount of cytosolic Nrf2 compared with cells treated with *t*-BHP alone. Previously, various studies demonstrated that candidate materials of chemopreventive agents can lead to the Nrf2 accumulation in nucleus and promoting of Nrf2-dependent gene expression [10, 34].

The change in the redox caused by oxidative stress is known to alter many signaling pathways, including MAPKs [35]. MAPK pathways mediated by ERK, JNK, and p38 have been demonstrated to play a central role in transducing extracellular signals to the nucleus [36]. Results from a study demonstrated that short-term treatment of rat prostate endothelial cells with *t*-BHP increased the level of p38 and ERK phosphorylation [37]. However, our result showed that HepG2 cells with *t*-BHP decreased JNK and ERK phosphorylation levels and that CA treatment activates these signaling pathways. To investigate the effect that MAPK phosphorylation has on gene expression, we probed HO-1 and GCL mRNA levels in the presence of specific MAPK inhibitors. In these experiments, we were able to observe that pretreatment with SB302580, a specific inhibitor of p38 or SP600125, a specific inhibitor of JNK for 1 h followed by treatment of 20 μM CA for 24 h, did not affect gene expressions; however, the mRNA levels of Nrf2, HO-1, GCLC, and GCLM significantly decreased as a consequence of pretreatment with the ERK-specific inhibitor PD98059 followed by treatment of CA suggesting that CA protects HepG2 cells against *t*-BHP-induced oxidative stress by activating Nrf2/ERK pathway. In our previous experiment, however, cells treated with CA (62.5–250 μM CA) for 1 h in the absence of *t*-BHP significantly (*p* < 0.05) increased phosphorylation of the JNK and c-Jun, whereas the phosphorylation of ERK and p38 was not affected [4]. And the pretreatment with PD98059 or SB203580 or SP600125 for 1 h followed by treatment of 250 μM CA for 1 h, did not affect gene expression of GCLM; however, the mRNA level of GCLC significantly (p < 0.05) decreased as a consequence of pretreatment with the JNK-specific inhibitor SP600125 followed by treatment of 250 μM CA indicating that CA increased GSH levels and GCLC in HepG2 cells via the JNK/AP-1 pathway. In a review article [38], regulation of expression of GCLC and GCLM subunit genes in HepG2 cells in response to a phenolic antioxidant β-naphtoflavonone are mediated by phorbol myristate acetate-responsive element (TRE/AP-1) and electrophile responsive element (its equivalent, ARE). Interestingly, activation of JNK and ERK also plays a role in antioxidant protection against *t*-BHP in the presence of CA, because HepG2 cells treated with SP600125 and with PD98059 displayed a significant decrease in cell viability (Fig. 6). Previously we demonstrated that phosphorylation of JNK and activation of the AP-1 transcription factor are associated with GCLC gene expression [4]. In the present study, we confirmed that treating cells with CA influenced GCLM gene expression as well as GCLC one via the ERK/Nrf2 pathway. This finding agrees with a recently study indicating that inhibition of ERK gene expression inhibited Nrf2-mediated induction of GCLM expression [39]. Therefore, HepG2 cell damage due to oxidative stress caused by *t*-BHP can be protected by CA treatment trough ERK/Nrf2 pathway as well as the JNK/AP-1 pathway to upregulate GCL expression leading to GSH synthesis.

## Conclusions

We have found that treatment of HepG2 cells with CA enhanced the expression of detoxification enzymes like HO-1, GCLC, and GCLM by way of ERK phosphorylation and Nrf2 activation. Thus, ERK/Nrf2 pathway influence the ability of CA in HepG2 cells to protect themselves against *t*-BHP-induced oxidative stress.

## Additional file

Additional file 1: **Table S1.** The cDNA sequences of primers for RT-PCR and qRT-PCR. (DOCX 17 kb)

## Data Availability

The datasets used and/or analyzed during the current study are available from the corresponding author upon reasonable request.

## Abbreviations

AP-1: Activator protein-1
ARE: Antioxidant responsive element
CA: Caffeic acid
DCFH-DA: Dichlorofluorescein diacetate
EMSA: Electrophoretic mobility shift assay
ERK: Extracellular signal-regulated kinase
FDNB: 1-fluoro-2,4-dinitrobenzene
GAPDH: Glyceraldehyde 3-phosphate dehydrogenase
GCL: Glutamate-cysteine ligase
GCLC: Glutamate-cysteine ligase catalytic subunit
GCLM: Glutamate-cysteine ligase modifier subunit
GSH: Glutathione
HO-1: Heme oxygenase-1
JNK: c-Jun N-terminal kinase
MAPKs: Mitogen-activated protein kinases
MEM: Minimum essential Eagle’s medium
MTT: 3-(4,5-dimethylthiazol-2yl)-2,5-diphenyl tetrazolium bromide
NEM: N-ethylmaleimide
NQO-1: NADP(H):quinone oxidoreductase-1
Nrf2: Nuclear factor-E2 p45-related factor
ROS: Reactive oxygen species
SOD: Superoxide dismutase
t-BHP: tert-butyl hydroperoxide

## References

[1] Wang C-J, Wang J-M, Lin W-L, Chu C-Y, Chou F-P, Tseng T-H (2000). Protective effect of Hibiscus anthocyanins against tert-butyl hydroperoxide-induced hepatic toxicity in rats. Food Chem Toxicol.

[2] Lin W-L, Wang C-J, Tsai Y-Y, Liu C-L, Hwang J-M, Tseng T-H (2000). Inhibitory effect of esculetin on oxidative damage induced by t-butyl hydroperoxide in rat liver. Arch Toxicol.

[3] Touaibia M, Jean-Francois J, Doiron J (2011). Caffeic acid, a versatile pharmacophore: an overview. Mini-Rev Med Chem.

[4] Yang SY, Kang JH, Seomun Y, Lee KW (2015). Caffeic acid induces glutathione synthesis through JNK/AP-1-mediated gamma-glutamylcysteine ligase catalytic subunit induction in HepG2 and primary hepatocytes. Food Sci Biotechnol.

[5] SIES H, SUMMER KH (1975). Hydroperoxide-metabolizing systems in rat liver. Eur J Biochem.

[6] Awe SO, Tsakadze NL, D'Souza SE, Adeagbo AS (2003). Tert-butyl hydroperoxide-mediated vascular responses in DOCA-salt hypertensive rats. Vasc Pharmacol.

[7] Garcia-Cohen E-C, Marin J, Diez-Picazo LD, Baena AB, Salaices M, Rodriguez-Martinez MA (2000). Oxidative stress induced by tert-butyl hydroperoxide causes vasoconstriction in the aorta from hypertensive and aged rats: role of cyclooxygenase-2 isoform. J Pharmacol Exp Ther.

[8] Ishii T, Itoh K, Takahashi S, Sato H, Yanagawa T, Katoh Y, Bannai S, Yamamoto M (2000). Transcription factor Nrf2 coordinately regulates a group of oxidative stress-inducible genes in macrophages. J Biol Chem.

[9] Itoh K, Chiba T, Takahashi S, Ishii T, Igarashi K, Katoh Y, Oyake T, Hayashi N, Satoh K, Hatayama I (1997). An Nrf2/small Maf heterodimer mediates the induction of phase II detoxifying enzyme genes through antioxidant response elements. Biochem Bioph Res Co..

[10] Ramos-Gomez M, Kwak MK, Dolan PM, Itoh K, Yamamoto M, Talalay P, Kensler TW (2001). Sensitivity to carcinogenesis is increased and chemoprotective efficacy of enzyme inducers is lost in nrf2 transcription factor-deficient mice. P Natl Acad Sci USA.

[11] Yang Y-C, Lii C-K, Lin A-H, Yeh Y-W, Yao H-T, Li C-C, Liu K-L, Chen H-W (2011). Induction of glutathione synthesis and heme oxygenase 1 by the flavonoids butein and phloretin is mediated through the ERK/Nrf2 pathway and protects against oxidative stress. Free Radical Bio Med..

[12] Feng J, Zhang P, Chen X, He G (2011). PI3K and ERK/Nrf2 pathways are involved in oleanolic acid-induced heme oxygenase-1 expression in rat vascular smooth muscle cells. J Cell Biochem.

[13] Lee H-S, Won NH, Kim KH, Lee H, Jun W, Lee K-W (2005). Antioxidant effects of aqueous extract of Terminalia chebula in vivo and in vitro. Biol Pharm Bull.

[14] Bissell DM, Hammaker LE, Meyer UA (1973). Parenchymal cells from adult rat liver in nonproliferating monolayer culture: I. functional studies. J Cell Biol.

[15] Janbaz KH, Saeed SA, Gilani AH (2004). Studies on the protective effects of caffeic acid and quercetin on chemical-induced hepatotoxicity in rodents. Phytomedicine..

[16] Duke JA. Handbook of phytochemical constituent grass, herbs and other economic plants. Boca Raton: CRC press; 1992.

[17] Taguchi K, Hagiwara Y, Kajiyama K, Suzuki Y (1993). Pharmacological studies of Houttuyniae-Herba - the Antiinflammatory effect of Quercitrin. Yakugaku Zasshi.

[18] Yang SY, Hong CO, Lee GP, Kim CT, Lee KW (2013). The hepatoprotection of caffeic acid and rosmarinic acid, major compounds of Perilla frutescens, against t-BHP-induced oxidative liver damage. Food Chem Toxicol.

[19] Park Ho-Young, Nam Mi-Hyun, Lee Hyun-Sun, Jun Woojin, Hendrich Suzanne, Lee Kwang-Won (2010). Isolation of caffeic acid from Perilla frutescens and its role in enhancing γ-glutamylcysteine synthetase activity and glutathione level. Food Chemistry.

[20] Kim MK, Lee HS, Kim EJ, Won NH, Chi YM, Kim BC, Lee KW (2007). Protective effect of aqueous extract of Perilla frutescens on tert-butyl hydroperoxide-induced oxidative hepatotoxicity in rats. Food Chem Toxicol.

[21] Yang SY, Hong CO, Lee H, Park SY, Park BG, Lee KW (2012). Protective effect of extracts of Perilla frutescens treated with sucrose on tert-butyl hydroperoxide-induced oxidative hepatotoxicity in vitro and in vivo. Food Chem.

[22] Kang JH, Yang SY, Ha J, Lee KW (2015). Perilla frutescens modulates CYP1A1/2 and HO-1 and activates Nrf2 in oxidative stress-induced hepatotoxicity. J Korean Soc Appl Bi.

[23] Nunes RG, Pereira PS, Elekofehinti OO, Fidelis KR, da Silva CS, Ibrahim M, Barros LM, da Cunha FA, Lukong KE, de Menezes IR, Tsopmo A, Duarte AE, Kamdem JP. Possible involvement of transcriptional activation of nuclear factor erythroid 2-related factor 2 (Nrf2) in the protective effect of caffeic acid on paraquat-induced oxidative damage in *Drosophila melanogaster*. Pestic Biochem Phys. 2019:161–68. 10.1016/j.pestbp.2019.03.017 31153464

[24] Pari L, Prasath A (2008). Efficacy of caffeic acid in preventing nickel induced oxidative damage in liver of rats. Chem Biol Interact.

[25] Ishii T, Itoh K, Sato H, Bannai S (1999). Oxidative stress-inducible proteins in macrophages. Free Radic Res.

[26] Kwak MK, Egner PA, Dolan PM, Ramos-Gomez M, Groopman JD, Itoh K, Yamamoto M, Kensler TW (2001). Role of phase 2 enzyme induction in chemoprotection by dithiolethiones. Mutat Res-Fund Mol M.

[27] Hayes JD, Chanas SA, Henderson CJ, McMahon M, Sun C, Moffat GJ, Wolf CR, Yamamoto M (2000). The Nrf2 transcription factor contributes both to the basal expression of glutathione S-transferases in mouse liver and to their induction by the chemopreventive synthetic antioxidants, butylated hydroxyanisole and ethoxyquin. Biochem Soc Symp.

[28] Wild AC, Moinova HR, Mulcahy RT (1999). Regulation of gamma-glutamylcysteine synthetase subunit gene expression by the transcription factor Nrf2. J Biol Chem.

[29] Mathers J, Fraser JA, McMahon M, Saunders RDC, Hayes JD, McLellan LI (2004). Antioxidant and cytoprotective responses to redox stress. Biochem Soc Symp.

[30] Pang C, Zheng Z, Shi L, Sheng Y, Wei H, Wang Z, Ji L (2016). Caffeic acid prevents acetaminophen-induced liver injury by activating the Keap1-Nrf2 antioxidative defense system. Free Radical Bio Med.

[31] Huang C-S, Lii C-K, Lin A-H, Yeh Y-W, Yao H-T, Li C-C, Wang T-S, Chen H-W (2013). Protection by chrysin, apigenin, and luteolin against oxidative stress is mediated by the Nrf2-dependent up-regulation of heme oxygenase 1 and glutamate cysteine ligase in rat primary hepatocytes. Arch Toxicol.

[32] Lv Hongming, Ren Hua, Wang Lidong, Chen Wei, Ci Xinxin (2015). Lico A Enhances Nrf2-Mediated Defense Mechanisms againstt-BHP-Induced Oxidative Stress and Cell Death via Akt and ERK Activation in RAW 264.7 Cells. Oxidative Medicine and Cellular Longevity.

[33] Shin Sang Mi, Yang Ji Hye, Ki Sung Hwan (2013). Role of the Nrf2-ARE Pathway in Liver Diseases. Oxidative Medicine and Cellular Longevity.

[34] Balogun E, Hoque M, Gong PF, Killeen E, Green CJ, Foresti R, Alam J, Motterlini R (2003). Curcurnin activates the haem oxygenase-1 gene via regulation of Nrf2 and the antioxidant-responsive element. Biochem J.

[35] Hsieh C-C, Rosenblatt JI, Papaconstantinou J (2003). Age-associated changes in SAPK/JNK and p38 MAPK signaling in response to the generation of ROS by 3-nitropropionic acid. Mech Ageing Dev.

[36] Su B, Karin M (1996). Mitogen-activated protein kinase cascades and regulation of gene expression. Curr Opin Immunol.

[37] Lee JY, Yu BP, Chung HY (2005). Activation mechanisms of endothelial NF-κB, IKK, and MAP kinase by tert-butyl hydroperoxide. Free Radic Res.

[38] Rahman I (2005). Regulation of glutathione in inflammation and chronic lung diseases. Mutat Res-Fund Mol M..

[39] Zipper LM, Mulcahy RT (2000). Inhibition of ERK and p38 MAP kinases inhibits binding of Nrf2 and induction of GCS genes. Biochem Bioph Res Co.

