# Supplementary material for: ERK/Nrf2 pathway activation by caffeic acid in HepG2 cells alleviates its hepatocellular damage caused by t-butylhydroperoxide-induced oxidative stress
Source: BMC Complement Altern Med. 2019 Jun 20;19:139. doi: 10.1186/s12906-019-2551-3 (PMC6585055; doi:10.1186/s12906-019-2551-3)
Supplement: Supplementary file 1 — Table S1. The cDNA sequences of primers for RT-PCR and qRT-PCR. (DOCX 17 kb) [file 12906_2019_2551_MOESM1_ESM.docx]

Table S1. The cDNA sequences of primers for RT-PCR and qRT-PCR

| Origin | Marker | Sequence | |
| --- | --- | --- | --- |
| Human | GCLC | Sense | 5′-AGT TGA GGC CAA CAT GCG AA-3′ |
|  |  | Antisense | 5′-TGA AGC GAG GGT GCT TGT TT-3′ |
|  | GCLM | Sense | 5′-ATC AAA CTC TTC ATC ATC AAC-3′ |
|  |  | Antisense | 5′-GAT TAA CTC CAT CTT CAA TAG G-3′ |
|  | HO-1 | Sense | 5′-GGA ACT TTC AGA AGG GCC AG-3′ |
|  |  | Antisense | 5′-GTC CTT GGT GTC ATG GGT CA-3′ |
|  | Nrf2 | Sense | 5′-AGA CAA ACA TTC AAG CCG CT -3′ |
|  |  | Antisense | 5′-CCA TCT CTT GTT TGC TGC AG-3′ |
|  | GAPDH | Sense | 5′-GCC ATC AAC GAC CCC TTC ATT-3′ |
|  |  | Antisense | 5′-CGC CTG CTT CAC CAC CTT CTT-3′ |
| Rat | GCLC | Sense | 5′-GAG TTC ATT GCA AAC CAT CCT GAC-3′ |
|  |  | Antisense | 5′-ACT CTG GAC ATT CAC ACA GCT CAT C-3′ |
|  | GCLM | Sense | 5′-TGT GTG ATG CCA CCA GAT TT-3′ |
|  |  | Antisense | 5′-GCT TTT CAC GAT GAC CGA GT-3′ |
|  | HO-1 | Sense | 5′-GAG CCA GCC TGA ACT AGC-3′ |
|  |  | Antisense | 5′-GAT GTG CAC CTC CTT GGT-3′ |
|  | β-actin | Sense | 5′-TCA GGA GGA GCA ATG ATC TTG A-3′ |
|  |  | Antisense | 5′-GAC AGG ATG CAG AAG GAG ATC AC-3′ |
